# American Medical Association at Detroit—Code of Ethics - Professor Sayre on Fractures

**Published:** 1874-07

**Authors:** 


### Our Exchanges.

---

*PHILADELPHIA MEDICAL TIMES—June*, 1874:

American Medical Association.—*First Day.*—The Association met June 2, at Hough's Theater, Detroit, at half-past eleven, Dr. J. M. Toner, President, being in the chair. After the opening prayer by Bishop McCoskry, Dr. William Brodie made a very neat speech of welcome, in which *inter alia*, he stated that when the Association first honored Detroit with its presence, eighteen years previously, the city numbered only forty thousand inhabitants; but now it boasts of a population of one hundred thousand, of a city hall that cost $600,000, and of a system of sewage so complete and so supplemented by other hygienic appliances and regulations that the mortality of the city in 1873 was only two and a half per cent.

President Toner then delivered his address, which contains much that is of interest, but is scarcely fitted for abstraction. We have failed to perceive any especial central idea in it, unless it be that of the necessity of progress, of the need of a first-class endowed physiological and pathological laboratory somewhere in the country, and of the general interest which the profession ought to feel in fostering original work.

Section 1.—Practice of Medicine, Materia Medica and Physiology. Dr. Bulkley, of New York, read a paper on "The management of Eczema," in which he treated at considerable length the various forms of local and constitutional cutaneous disease. Among the remedies applied by him in his practice, with varied failures and successes, were arsenic, glycerin, cod-liver oil, tar ointment, mutton-suet and lard, carbolic acid, and a solution of tar and caustic potash.

Dr. Farnsworth, of Iowa, then read a long paper on "Ammonia," most of which is only of interest, first, as showing how much easier it is to speculate and reason than to work out new facts in the laboratory; secondly, as revealing of how little permanent value work of the former kind is when done. Although ammonia is in so frequent use we have almost no positive knowledge as to its action, and Dr. Farnsworth's paper throws no light on the subject.

SEC. 2.—Obstetrics. There does not appear to have been anything of much interest before this section.

SEC. 3.—Surgery and Anatomy.

Dr. Dunlap read a well-written report of a case of Enchondroma over the Sternum.

Dr. E. M. Moore then read a paper on the Epiphyseal Fracture of the Humerus. * * *

Diagnostic points are, first, the projection beneath the coracoid; and second, the immediate recurrence of the deformity when the means for reduction cease retaining the shaft in place, there being no fracture of the superior end of the humerus in which retention is so difficult. In general the true nature of the injury is unrecognized, although the symptoms have been clearly stated by Sir A. Cooper and Professor R. W. Smith and Frank H. Hamilton. This arises from no accurate conception of the change of position having been put forth, and no method securing precise retention having been proposed, in so far as he (Dr. Moore) was aware. * * *

Reduction is effected by carrying the arm forward and upward to the perpendicular line. Retention is effected by moderate extension while bringing the arm down by the side, maintaining this slight extension until dressings for the purpose of continuing it are applied. Swinburne's method fulfils the indications easily and perfectly. Even if not restored the arm soon becomes useful, and nature gradually rounds off the prominence of the diaphysis and elongates the capsule at the lower border, allowing the motion upward to improve. * * *

Dr. Lewis A. Sayre, of New York City, had been appointed to make a report on fractures, two years ago, but was prevented by illness. He presented statistics taken from Bellevue Hospital, prepared by House-Surgeon Van Wagner. He had lately learned of actual cases really treated by what he had supposed to be the obsolete method of waiting nine days for the swelling of the soft parts to subside. Again, most authorities on fracture stated that in fractures of the long bones, particularly if oblique, we were to expect deformity. Some say it is quite impossible without it. He thought this wrong, and the sooner corrected the better. Statistics proving shortening should only be saved to be disentombed to assist in defeating suits for malpractice. The long bones keep the body in place. Fracture occurring, the muscles contract and produce distortion of the vessels and nerves, the ends of the fragments causing irritation and inflammation. All that is necessary in any fracture is extension and counter-extension in the right direction, so as to have accurate adjustment. * * *

Dr. Sayre stated that he had said that extension and counter-extension should be made in the proper direction, and then on no side would any muscle be stretched. All he had claimed was that it was better to start out with the idea that we could secure perfect results rather than that we must necessarily have shortening. * * *

Dr. Gregory, of St. Louis, expressed his astonishment. He thought Dr. Sayre's results barely possible, and wanted further evidence. He treated his cases by extension and counter extension, with all the perfection of adjustment possible with mechanical appliances. If he had seen fractures occur without shortening he had forgotten it. * * * Inflammation always consumes some of the soft parts. A young person's getting no shortening might be accounted for by irritation; but he did not think an adult could get union without shortening. Again, precise adjustment was practically impossible: the reverse was all talk, and might do to tell *students.* * * *

Sec. 4—Medical Jurisprudence, met only to adjourn.

Sec. 5—Public Hygiene, was engaged in a long discussion upon the propriety of petitioning Congress to establish a national health bureau, and finally decided in the affirmative, and also adopted the following resolution:

"*Resolved*, That, with a view to the establishment of a national sanitary bureau, it is expedient, at the present time, to press, through State medical societies and physicians everywhere, upon the legislatures of the several States the importance of establishing State boards of health."

### SECOND DAY.

The meeting was called at the Opera House at 9.30 A. M. After the announcement of the Committee on Nomination, and the election of permanent members, several resolutions of no general interest were acted upon.

Dr. N. S. Davis then read the report of the Judicial Council. The report was adopted on the motion of Dr. Vandeman, of Tennessee. It stated that letters had been addressed to thirty or forty persons in different parts of the country, and of these only five expressed a decided dissatisfaction with the present code, whilst fourteen were positively opposed to the slightest alteration; an evidence that the great majority of the profession are satisfied with the code as it is.

After a careful examination of the code, the Council is of the opinion that it expresses the ethical relations and duties of the profession as completely and concisely as possible.

In regard to specialties, the Council report is *verbatim* as follows:

"Then the code of ethics very properly makes no mention of specialties or specialists, but presents plainly the rules necessary for the maintenance of professional character as applicable to all. But we are asked, How, then, can those who wish to pursue a special practice make known their position to their brethren and the public? We answer that the title of Doctor of Medicine covers the whole field of practice, and whoever is entitled to that appellation has the right to occupy the *whole* or any part of the field, as he pleases. The acceptance of this honorable title is presumptive evidence to the community that the man accepting it is ready to attend practically to any and all duties which it implies. As all special practice is simply a self-imposed limitation of the duties implied in the general title of doctor, it should be indicated, not by special or qualifying titles, such as *oculist, gynæcologist*, etc., nor by any positive setting forth of special qualifications, but by a simple, honest notice appended to the ordinary card of the general practitioner, saying: 'Practice limited to diseases of the eye and ear,' or 'to diseases peculiar to women,' or "to midwifery exclusively,' as the case may be. Such a simple notice of limitation, if truthfully made, would involve no other principle than the notice of the general practitioner that he limits his attention to professional business within certain hours of the day. Neither could it be regarded as a claim to special or superior qualifications. To give the specialist any privilege beyond this would be to invest him with a special privilege inconsistent with the equality of rights and duties pertaining to the whole profession. We see no reason, therefore, for recommending any change in the present code of ethics in reference to this subject."

In regard to doctors bidding against one another for public services, the report says:

"The present code of ethics, while sanctioning a most liberal bestowal of gratuitous professional service to the poor, whether as individuals or in public charitable institutions, and in aid of the sanitary interests of the communities, yet expressly prohibits the bestowal of such services on well-to-do individuals, endowed, mutual benefit, or any kind of money-making institutions, societies or corporations. It also expressly prohibits all attempts to attract attention and make merchandise of charity by ostentatiously parading before the public notices proffering services and medicine to the 'poor gratis.' We see no reason why this is not sufficient, so far as relates to the regulation of gratuitous services. To govern the matter of compensation the code simply gives us the following general declaration: 'Some general rules should be adopted by the *faculty*, in every town or district, relative to *pecuniary acknowledgments* from their patients; and it should be deemed a point of honor to adhere to those rules with as much uniformity as varying circumstances will admit.' The aim appears to have been to allow sufficient variations in the rate of compensation to accommodate the varying habits and circumstances of different communities, and yet to bind each individual to an honorable compliance with the general rules established by his professional brethren. Such being the correct ethical principle, the difficulty consists in tracing and maintaining clearly its practical application. That the principle laid down in the paragraph just quoted is inconsistent with all contracts or agreements to attend individuals, families, companies, corporations, or any associations or institutions other than those of a strictly charitable character, for a specified sum per month or year, without regard to the amount of medical services that might be required in the time specified, no one can reasonably doubt. It seems to us equally inconsistent with the ethical rule to enter into a contract with a manufacturing company to attend their employees, or with a school to attend its patrons or scholars, for a fixed sum per annum, to be derived from the levy of a certain percentage on the wages of the employees or on the tuition fees of the students; for, however plausible may be the humanitarian idea of securing for the employee and the student adequate medical attendance when sick at the smallest average cost, the practical working of the system violates both the rule that compensation for medical services should be in accordance with the kind and amount of services rendered, and that every individual and family should be free to choose their own medical attendant without dictation or indirect restraint.

"These observations do not apply to a certain kind of contract service sometimes required in connection with the medical staffs of the army and navy, nor to the hospital tax on sailors in the marine hospital system, for reasons too obvious to require mention. One other subject requires a few moments attention. There is a class of public charitable institutions, such as county alms-houses, ophan asylums, etc., supported by public taxation. In many of the States the public authorities having control of such institutions have annually asked for bids from the profession, offering to award the contract for professional services to the one who should bid for the lowest pecuniary consideration.

"While as charitable institutions any member of the profession might offer his services to such of the poor inmates as might ask for them gratuitously, yet the idea of asking members of the profession to bid against each other for the pay for public professional services is repugnant to every feeling of professional honor, and often productive of great injustice to the sick poor.

"The public authorities in all such cases should fix such just rate of compensation for the necessary medical services as they may deem best, and then appoint the best medical man who is willing to accept the compensation proposed. And we have no doubt but that a proper attention to this subject on the part of the profession would secure the necessary change."

The amendments of the plan of organization next came up. After considerable discussion, the following amendment was adopted:

Strike out the second paragraph of Article II, and insert the following:

"The delegates shall receive their appointment from permanently organized State medical societies, and such County and District medical societies as are recognized by representation in their respective State societies, and from the medical department of the army and navy of the United States."

Also, strike out the fourth paragraph of same article, and insert:

"Each State, County and District medical society, entitled to representation, shall have the privilege of sending to the Association one delegate for every ten of its regular resident members, and one for every additional fraction of more than half that number. Provided, however, that the number of delegates from any particular State, territory, county, city, or town, shall not exceed the ratio of one in ten of the resident physicians who may have signed the code of ethics of this Association.

"The medical staffs of the army and navy shall be entitled to four delegates each."

Dr. N. S. Davis then delivered the annual address upon Practical Medicine. This, although containing food for thought, is long, and has not enough of novelty to warrant its reproduction.

Dr. Gross' address on Surgery was confined chiefly to a consideration of the subject of prosecution, and has not yet been reported.

Section 1—Practice of Medicine, Materia Medica and Physiology. About a dozen members present. A report from the Committee on the Cultivation of the Cinchona Tree was read, and the committee continued, in accordance with their own request.

A very long and elaborate article on The Mechanism of the Encephalic Circulation, by Dr. Reuben A. Vance, of New York, was read.

Sec. 2—Obstetrics. A paper upon the Inverted Uterus, by Dr. Bontecue, of Troy, New York, was read and referred to the Publication Committee.

Sec. 3—Surgery. The discussion of Dr. Sayre's paper on Fractures was resumed.

Dr. A. Garcelon, Secretary of the Section, spoke of the great practical value of the question to the profession, and said that while he thoroughly agreed with Dr. Sayre in striving to secure a perfect limb in cases of fracture, and also in aiming at a perfectly accurate adjustment, his own experience showed him the difficulty of succeeding in the latter attempt, and he believed that *no distinct and definite rule* of treatment could be laid down which would show such a series of results as were given in the statistics submitted by Dr. Sayre, for they show a most wonderful absence of shortening.

Dr. Gross said he used in preparing his bandages a solution of silicate of potash and silicate of soda, to which he gave a preference over plaster of Paris, and a decided preference over starch, to which he was strongly opposed. He said it dried very rapidly. On the main question as to whether there was necessarily shortening in oblique fractures of the femur, he was in favor of the view that a certain amount of shortening was necessary.

Dr. Reed, of Ohio, thought that in every case there must be shortening, and he took the ground that in cases of apparent absence of this defect nothing could be known to a certainty. * *

Dr. Whiting, of Wisconsin, had been surprised at the position taken by Dr. Sayre; and the idea that the theory of the necessary shortening of the long bones in cases of oblique fracture was exploded, alarmed him. * * *

Dr. Quimby, of Jersey City, did not believe that any surgeon thought himself able to restore ten fractured femurs in a hundred to their original length; that an absolutely accurate measurement was practically impossible, and it is scarcely ever that one finds an exact restoration. The proposition of a perfect cure seemed to him too absurd to require remark. He had seldom seen a case of less than one-half inch shortening.

Dr. Hughes, of Iowa, thought there were but few present who would agree with Dr. Sayre. He did not question the veracity of the statistics, but if they were true he considered them simply wonderful. * * *

Dr. Waterhouse, of Wisconsin, said that if these statements are true, and these figures accurate, we should face the situation, and let the danger of suits come.

There is a reason for this unheard of success at Bellevue in the appliances at hand, and the constant attendance and care available, which country practitioners can never enjoy; and suits for malpractice are generally brought in the country. Backwoods doctors scarcely every have proper appliances, and cannot possibly give cases constant personal care. * * *

Dr. Pierce, of Illinois, thought the facts should be known, and if surgery has made such advances it should be declared.

Dr. Sayre, of New York, said he was glad to have been the means of stirring up a discussion on this subject, but he saw he had been *grossly* misunderstood.

A voice.—"No reference to the chair, is there, doctor." [Laughter.]

Dr. Sayre.—"The chair never misunderstands." [Applause.]

After this little episode the doctor said that he *knew* the statistics presented by himself to be correct; that Dr. Hamilton had made the measurements, and that he was a man who was so violently opposed to the theory that in his published writings he had denied the possibility of any oblique fracture being cured without shortening. For this reason he (Dr. Sayre) had asked him to measure the patients. He said that if seven successive cases should be presented he would agree to give up his opposition to the theory. He found the cases and surrendered.

Dr. Sayre now turned and said, "Did Dr. Gregory say yesterday that it was impossible?"

Dr. Gregory answered, "Yes."

Dr. Sayre then said he was glad to have found an authority at last; that the reasons he had concluded to make a report were, as he stated on Tuesday, because he had heard of practitioners who clung to obsolete theories, and now he was glad to find that there had been a necessity for his doing so. He then read an article from the *Medical Journal*, signed by Dr. Hamilton, sustaining his position. He said the credit of the results at Bellevue is not due to the attending surgeons, but to the young men of the institution, who make the preliminary arrangements and have the care of the patients.

Dr. Sayre then took up the mode of treatment he had advocated on Tuesday, produced his machine of wood and rubber, and briefly repeated his theory in regard to extension and counter-extension. He claimed that there is never irritation of the muscle when there is only normal traction. If there is only enough extension to produce a normal condition, then there is retention—*fixation*. Too much extension is abnormal, produces irritation, and is often the cause of non-union.

Extension and counter-extension should be applied the instant of seeing the patient, and then—fixation. There are no circumstances when something cannot be found to effect this. Machinery is the curse of the profession, and there is scarcely a country doctor who has not in his office a lot of worthless and expensive machines called "surgical appliances."

In setting a limb, the assistant who holds it in position while the surgeon is applying the bandages is in the most responsible position. If there is a single continuous piece of skin (no matter how crooked or narrow) for a guide, then the limb can be put in position, be held by an assistant, and properly attended to.

The speaker said that in thigh-fractures he used the triple inclined plane. He disclaimed any desire to "lay down the law," and concluded by claiming that a discussion of this kind would not have the authority of a carefully written book.

Dr. Lockford, of Missouri, believed with Dr. Sayre that perfect cures can be effected, and that the profession should strive for perfection.

Dr. King, of Philadelphia, at the request of Dr. Sayre, took the floor, and stated that he fractured his thigh over a year ago, and that now there was no apparent and only a very slight actual shortening of the limb.

The discussion was continued some time longer, after which Dr. King moved that Dr. Sayre's paper be referred to the Committee on Publication.

Dr. Quimby, of Jersey City, moved to substitute for Dr. King's motion, the following preamble and resolution:

*Whereas,* There seems to be some uncertainty in the minds of the profession in reference to their treatment of fractures of the long bones, therefore,

*Resolved,* That it is the opinion of this section that it is nearly impossible to treat fractures without shortening.

The substitute was lost, and the paper referred to the Committee on Publication.

THIRD DAY.

The Association met punctually at 9.30 A.M.

Suitable resolutions in regard to the death of the late Dr. Henry Miller were read and adopted.

An appeal from the Boyle County Medical Society, of Kentucky, was read by Dr. Keller, and the following preamble and resolutions, after discussion, were adopted:

*Whereas,* A most laudable effort has recently originated in the Boyle County Medical Society, of the State of Kentucky, and in the Kentucky State Medical Society, to create a fund for the erecting of a statue or some other suitable memorial in honor of Dr. Ephraim McDowell, "the father of ovariotomy," English writers to the contrary notwithstanding, who lived in the town of Danville, in the State of Kentucky, and who performed in that town the first ovariotomy, in the year 1809:

*Resolved,* That the American Medical Association most earnestly endorse the action of said County and State Societies, and as urgently commend the object to the generous consideration of the medical profession of the world.

The Committee on Prize Essays reported that none, in their estimation, worthy of the prize had been offered.

A report from Dr. Seguin concerning his visit abroad as representative of the American Medical Association was next read. * * * It concludes as follows:

"These considerations could be much enlarged. To save time, I cut them short, and conclude by asking the American Medical Association to form an international commission of physicians of high standing, speaking the European languages, and designing to go to Europe this summer.

"To charge them to represent the American Medical Association before the British Medical Association, the German and French Associations for the Advancement of Science, and other kindred societies; to promote interchange of ideas, and particularly to co-operate with them in any plan, scheme, or organization which would have for direct object the uniform establishment and propagation of the methods and instruments of positive observation. And, finally, to invite them to report on the progress of this work at the next meeting of this Association."

A proposal to amend the plan of organization was submitted by Dr. Adams Jewett, of Ohio:

"The permanent members shall consist of all those who have served in the capacity of delegates, and of such other members as shall have received the appointment by unanimous vote, and of all others who being members in good standing of any State or local medical society entitled to representation in this body shall, after being vouched for by at least three members, be elected to membership by a vote of three-fourths of the delegates in attendance, and shall continue such so long as they remain in good standing in the body of which they were members when elected to membership in this Association, and comply with the requirements of its by-laws."

This was laid over for one year.

Sec. 1. Dr. L. D. Bulkley read an article on "A New Antipruritic Remedy," which was referred to the Committee on Publication. The principal facts in Dr. Bulkley's paper are as follows:

Eighteen months ago he presented to the profession the *liquor picis alkalinus*, which had proved itself valuable in certain cutaneous affections in relieving itching in many instances, and whose value in this and other directions is daily becoming more evident. The failure of this as an antipruritic in some cases, as might be expected, led him to the adoption of the compound presented, which had thus far rendered inestimable service, but which had not to his knowledge been heretofore known. A remedy formed of hydrate of chloral and gum camphor, equal parts, rubbed well together, was found to be convertible into a transparent, colorless fluid, of the consistency of glycerin. It at once occurred to Dr. B. that it would be of value in cases presenting the above symptoms, and he had long employed the chloral internally with good results. He had some ointment prepared of pulverized gum camphor, hydrate of chloral, and rose ointment, which produced excellent effects when rubbed on the healthy skin. The compound was also soluble in almond oil, alcohol, and ether. Two cases were cited in which the remedy had been employed with satisfactory results.

Dr. Garrish, of New York, then read a long paper upon hydrophobia, containing an interesting history of the disorder, but nothing at all novel, unless it be the suggestion that bronchotomy should be resorted to in the treatment of the disorder.

Sec. 3. Dr. Beard made an extended address upon the use of electricity, in which he arrived at the following conclusions:

1. That certain benign tumors, as goitres, cystic tumors, and nævi, can be made to diminish or to disappear under electrolysis.

2. That fatty tumors and many enlarged glands usually do not much diminish under electrolysis, and sometimes will not diminish at all.

3. That malignant tumors will not usually diminish, and rarely, if ever, disappear, under electrolysis; but the pain connected with them can be treated most successfully, not only by electrolysis, but also by simple external electrization.

4. That malignant tumors, when sufficiently accessible and not too far advanced, may be treated by the method of *electrolizing the base, or working up the base,* as Dr. B. terms it, and this method promises more permanent results than have been obtained by the usual treatment.

5. That certain diseases of the skin—herpes, eczema, and prurigo—may be treated by different methods of using electricity with the highest success.

6. That diseases of the skin may be treated by local and *central* methods of using electricity; but some of the most brilliant results in the treatment of chronic eczema have been obtained by galvanizing the nerve-centres, in the method of *central galvanization,* without making any application whatever to the diseased parts. The results of this method of treatment seem to show pretty conclusively that chronic eczema is to a considerable extent dependent on the central nervous system.

The question of fractures was again brought up.

Dr. J. W. Hughes, of Pennsylvania, stated that the action of the Section in reference to the rejection of Dr. Quimby's motion in reference to Dr. Sayre's paper upon fractures looked better for Dr. Sayre than it did for the gentlemen present, a large majority of whom were undoubtedly opposed to the theories advanced and sustained by the statistics furnished. He therefore, for the purpose of placing the Section right before the profession, offered the following:

*Whereas*, The members of the Surgical Section of the American Medical Association have listened with interest to the report of Prof. Sayre, of New York, on the subject of fractures, and whereas, statistics accompanying said report evince in the institution represented unusual results; therefore,

*Resolved*, That the Section, after free discussion of the report, and its reference to the Publishing Committee, would express their opinion, based upon experience, that the results in relation to shortening following fractures, are better than can be looked for in general practice.

Adopted.

Dr. William P. Pierce, of Lamont, Illinois, offered the following, which was adopted:

*Resolved*, That this Section hereby requests of Prof. Sayre that as far as possible he should cause a second measurement to be made of all these cases, and report the result of such measurement at the next session of this Association.

In the afternoon session Dr. D. O. Farrand, of Detroit, introduced two cases of oblique fracture of the femur in boys, which he claimed to have cured, one with only a very slight shortening, the other with absolutely none, and asked those interested to examine the boys. The larger of the two was ten years old; his fracture had taken place six years previously, and he it was whose cure was said to be perfect. He was taken into the jury room, undressed, and, on motion of Dr. Sayre, was measured by a committee of all who did not believe in the completeness of the cure. Nearly every one present examined him, and a number of measurements were taken, some finding the leg which had been broken to be the shorter, while others claimed the contrary. Among those who stated that absence of shortening was totally impossible was Dr. J. T. Hodgin, of St. Louis, who was apparently much chagrined when he reported that the left leg was the *shorter*, when it turned out that the right was the one which had been broken. He, however, claimed a triumph, as he said the result only showed the accuracy of his position "that a correct measurement was an impossibility."

Dr. Sayre was evidently delighted at this evidence in favor of his theory of the possibility of perfect cures, and not only that, but that it also went to sustain him in his statement that the younger members of the profession were the most successful men.

SEC. 4. Papers were read by Dr. E. Lloyd Howard, of Baltimore, on Emotional Insanity, and by Dr. A. N. Talley, on the Relations of Psychology to Medicine, and were referred to the Publication Committee.

SEC. 5. Various matters were discussed, the most important being as follows:

A resolution offered by Dr. H. B. Baker, of Lansing, Michigan, was adopted. Under it Drs. Baker, H. A. Johnson, of Chicago, and Toner, of Washington, were appointed a committee to draft a bill for the establishment of a National Council of Health at Washington, and otherwise to forward the proposed measure.

Dr. Horner offered his annual resolutions in regard to alcohol, and, after a long discussion, had the satisfaction of having them passed, sixteen members being present.

FOURTH DAY.

The Asssociation was called to order at the usual hour.

Dr. H. T. Byford, of Colorado, submitted the report of the Committee on Nominations, which was adopted, as follows:

*President*—Dr. W. K. Bowling, Tennessee.

*Vice-Presidents*—1. Dr. William Brodie, of Michigan; 2. Dr. J. J. Woodward, of United States Army; 3. Dr. H. W. Brown, of Texas; 4. Dr. H. D. Didama, of New York.

*Treasurer*—Dr. Caspar Wister, of Pennsylvania.

*Librarian*—Dr. William Lee, of District of Columbia.

*Committee on Library*—Dr. Johnson Elliott, of District of Columbia.

*Assistant Secretary*—Dr. Will. Walling, of Kentucky.

*Committee of Arrangements*—Drs. Edward Richardson, Chairman; Lawrence Smith, Robert Gale, James Holland, Henry Bullitt, J. M. Keller, D. W. Yandell, Lewis Rogers, R. C. Hewett, all of Louisville.

*Committee on Prize Essays*—Drs. J. A. Octerloney, L. P. Yandell, J. D. Jackson, all of Kentucky; Theophilus Parvin, T. M. Stevens, both of Indiana.

*Committee of Publication*—Drs. F. G. Smith, Wm. B. Atkinson, D. Murray Cheston, Caspar Wister, Alfred Stille, all of Pennsylvania; William Lee, of District of Columbia; H. F. Askew, of Delaware.

Next place of meeting, Louisville, Kentucky.

Time of meeting, first Tuesday in May, 1875.

The committee also report the following nominations for Chairmen and Secretaries of Sections for 1875:

1. Practice of Medicine, Materia Medica, and Physiology, Dr. Austin Flint, of New York, Chairman, and Dr. J. K. Bartlett, of Wisconsin, Secretary.

2. Obstetrics and Diseases of Women and Children, Dr. W. H. Byford, of Illinois, Chairman, and Dr. S. C. Bussey, of District of Columbia, Secretary.

3. Surgery and Anatomy, Dr. E. M. Moore, of Rochester, New York, Chairman, and Dr. Thomas S. Lattimer, of Maryland, Secretary.

4. Medical Jurisprudence, Chemistry, and Psychology, Dr. Jerome Cochran, of Alabama, Chairman, and Dr. G. A. Moses, of Missouri, Secretary.

5. State Medicine and Public Hygiene, Dr. H. I. Bowditch, of Massachusetts, Chairman, and Dr. H. B. Baker, of Michigan, Secretary.

*Judicial Council*—Drs. J. K. Bartlett, Wisconsin; R. H. Gale, Kentucky; J. B. Johnson, Missouri; J. R. Bronson, Massachusetts; B. H. Catlin, Connecticut; Franklin Staples, Minnesota; W. T. Briggs, Tennessee, in place of the seven whose terms expire at this meeting. Dr. A. N. Talley, of South Carolina, for two years, to fill vacancy. The rest of the present council continued.

Dr. Keller, of Kentucky, offered the following resolution, which was adopted:

*Resolved*, That in furtherance of the views expressed by Dr. Gross in his valuable address touching a proper legislation to prevent the spread of syphilis, a committee composed of Dr. Gross, Dr. N. S. Davis, Dr. J. M. Toner, Dr. Marion Sims, and

Dr. John Morris, be appointed to report at the next meeting the most feasible plan for securing such legislation.

A paper by Dr. Paul F. Eve, on Surgery in the West was read.

